# Supplementary material for: Distinct changes in endosomal composition promote NLRP3 inflammasome activation
Source: Nat Immunol. 2022 Nov 28;24(1):30–41. doi: 10.1038/s41590-022-01355-3 (PMC9810532; doi:10.1038/s41590-022-01355-3)
Supplement: Supplementary file 1 — Supplementary Figs. 1–5, unprocessed western blots of Supplementary Figures. [file 41590_2022_1355_MOESM1_ESM.pdf]

nature immunology

Article

https://doi.org/10.1038/s41590-022-01355-3

Distinct changes in endosomal composition promote NLRP3 inflammasome activation

In the format provided by the authors and unedited

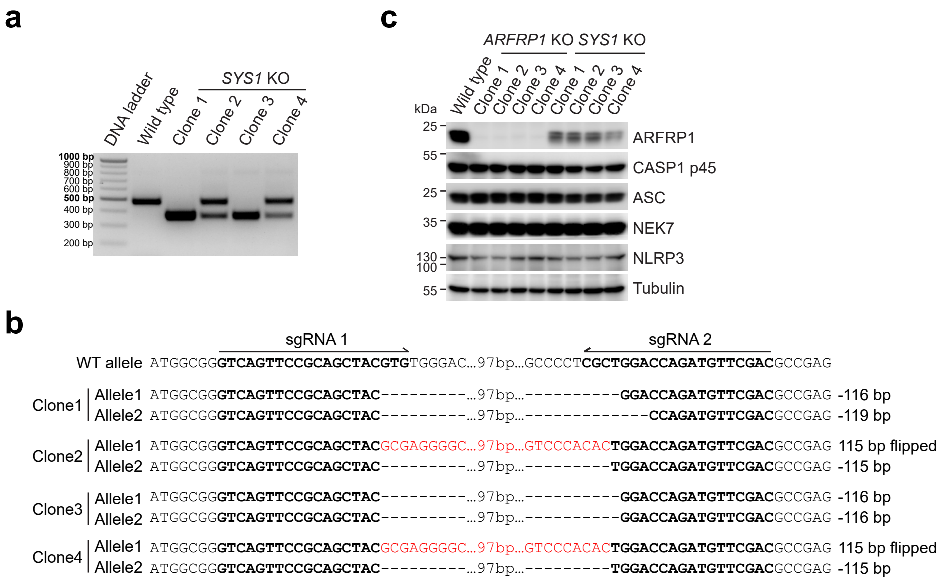

Supplementary Figure 1 Validation of *ARFRP1* KO and *SYS1* KO THP-1 cells generated by CRISPR/-Cas9-mediated gene editing. a, Genotyping of *SYS1* KO THP-1 cell clones by PCR. **b**, Validation of THP-1 *SYS1* KO clones by Sanger sequencing. **c**, Immunoblotting of cell lysates from wild type (WT), *ARFPR1* KO and *SYS1* KO THP-1 cells. Antibodies against ARFRP1, CASP1, ASC, NEK7 and NLRP3 were used. An antibody against Tubulin was used as a loading control. Data shown in panel a and c are representative of three independent experiments. Data shown in panel b was performed once.

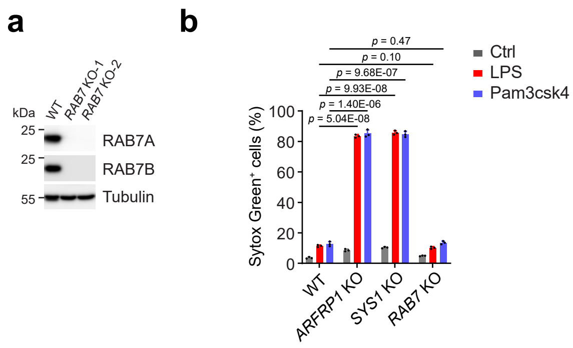

Supplementary Figure 2 Deletion of *RAB7* did not potentiate NLRP3 inflammasome activation. **a**, Immunoblotting of cell lysates from wild type (WT) and *RAB7* KO THP-1 cells. Antibodies against RAB7A and RAB7B were used. An antibody against Tubulin was used as a loading control. **b**, Cellular uptake of Sytox Green in wild type (WT), *ARFRP1* KO, *SYS1* KO and *RAB7* KO (*RAB7* KO-1) THP-1 cells treated with vehicle (Ctrl), 1 µg/ml LPS or 1 µg/ml Pam3csk4 for 2 hours. Means ± SD, N=3. Data were analyzed with an unpaired two-sided *t*-test (b). Data shown in a are representative of three independent experiments.

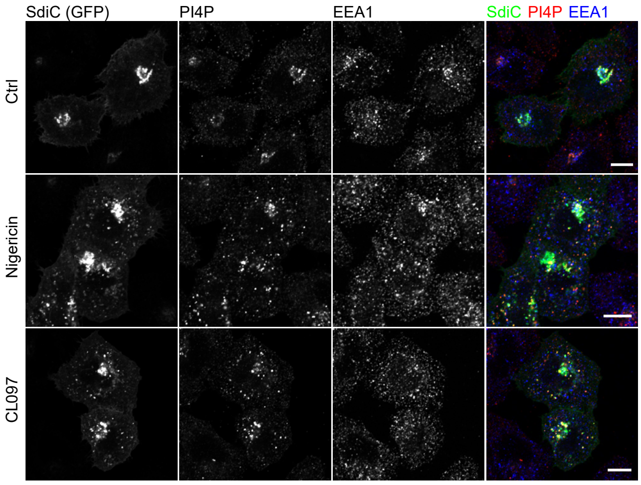

Supplementary Figure 3 Visualization of intracellular PI4P by indirect immunofluorescence and SidC-GFP. Indirect immunofluorescence using an anti-PI4P antibody and the PI4P probe SidC-GFP showed similar distribution pattern of intracellular PI4P. HeLa cells expressing SidC-GFP were treated with vehicle (Ctrl), 15 μM nigericin or 45μg/ml CL097 for 60 min. After fixation, cells were co-stained with antibodies against PI4P and EEA1. Scale bar: 10 μm. Data shown are representative of three independent experiments.

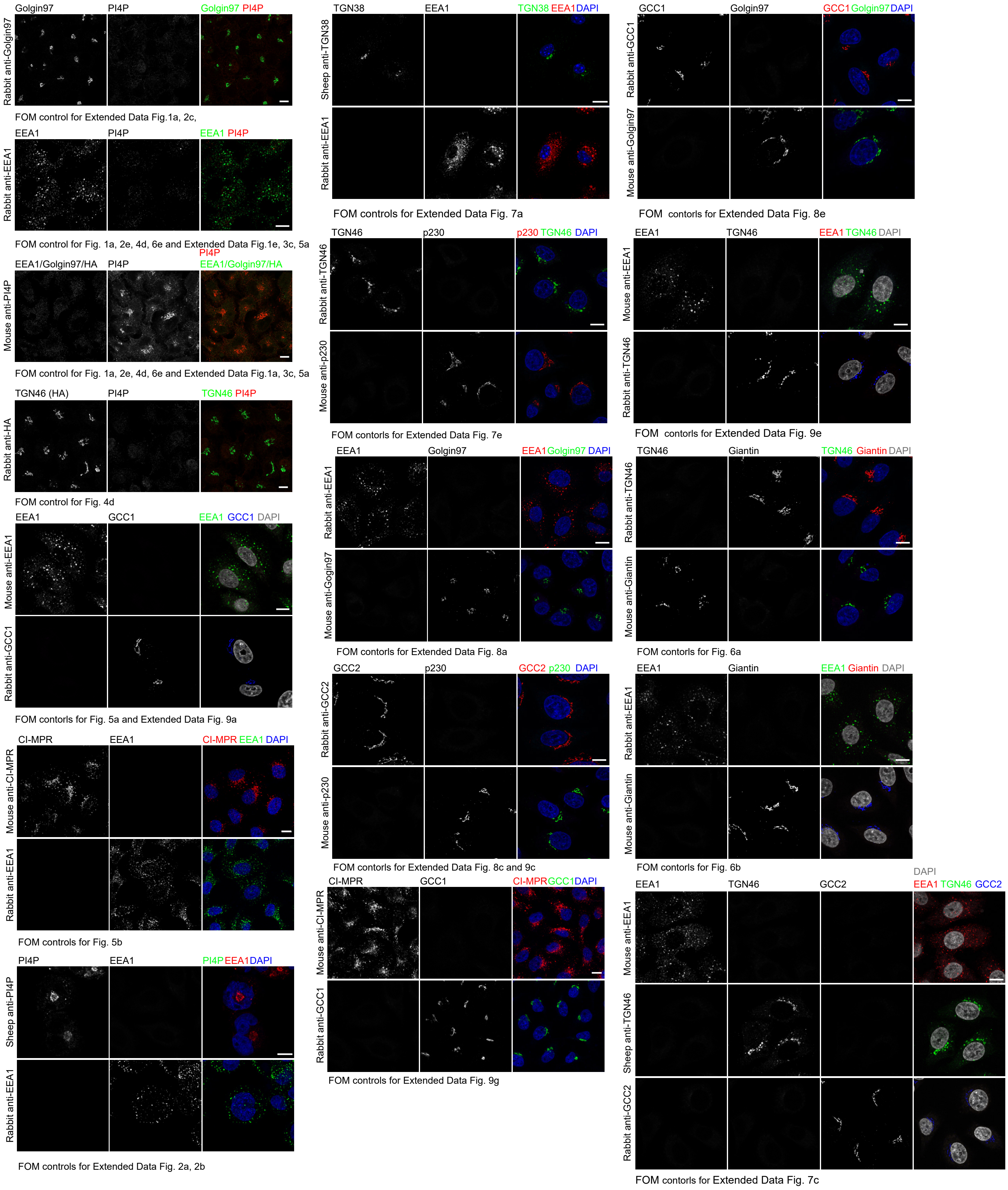

Supplementary Figue 4 Fluorescence-minus-one (FMO) controls for all fluorescently labelled antibodies. Cells were co-stained with indicated antibody and control IgG/IgM from the same specie of cognate antibody. Scale bar: 10 μm.

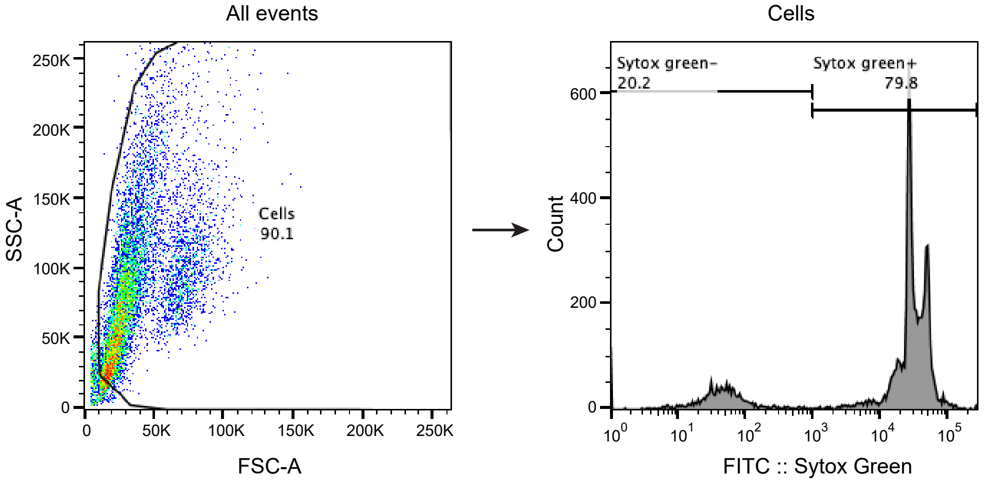

Supplementary Figue 5 The gating strategy of FACS analysis of pyroptotic cell death by Sytox green staining

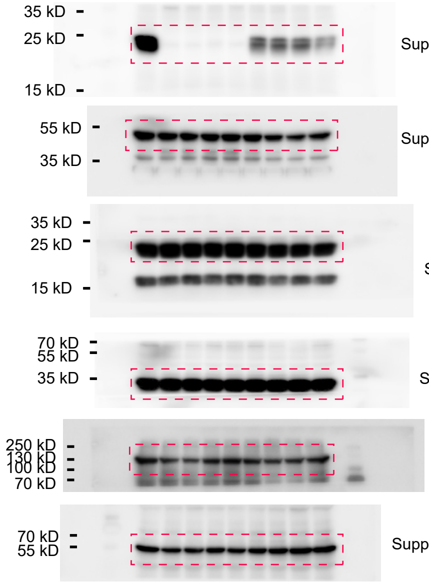

Supplementary Figure 1c_ARFRP1

Supplementary Figure 1c_CASP1

Supplementary Figure 1c_ASC

Supplementary Figure 1c_NEK7

Supplementary Figure 1c_NLRP3

Supplementary Figure 1c_Tubulin

Unprocessed Western Blots of Supplemenatry Figure 1c

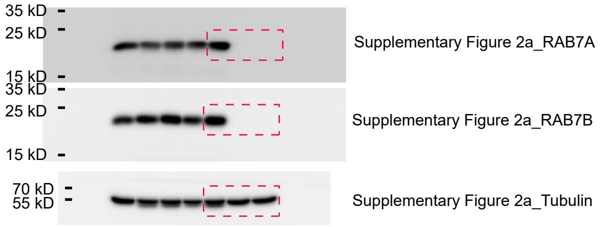

Unprocessed Western Blots of Supplemenatry Figure 2a
