# Supplementary material for: Distinct changes in endosomal composition promote NLRP3 inflammasome activation
Source: Nat Immunol. 2022 Nov 28;24(1):30–41. doi: 10.1038/s41590-022-01355-3 (PMC9810532; doi:10.1038/s41590-022-01355-3)
Supplement: Supplementary file 2 — Reporting Summary [file 41590_2022_1355_MOESM2_ESM.pdf]

nature portfolio

Corresponding author(s): Antonella de Matteis, Zhirong Zhang and Romeo Ricci

Last updated by author(s): Sep 1, 2022

Reporting Summary

Nature Portfolio wishes to improve the reproducibility of the work that we publish. This form provides structure for consistency and transparency in reporting. For further information on Nature Portfolio policies, see our Editorial Policies and the Editorial Policy Checklist.

Statistics

For all statistical analyses, confirm that the following items are present in the figure legend, table legend, main text, or Methods section.

n/a	Confirmed	
☐	☒	The exact sample size (*n*) for each experimental group/condition, given as a discrete number and unit of measurement
☐	☒	A statement on whether measurements were taken from distinct samples or whether the same sample was measured repeatedly
☐	☒	The statistical test(s) used AND whether they are one- or two-sided<br>*Only common tests should be described solely by name; describe more complex techniques in the Methods section.*
☐	☒	A description of all covariates tested
☒	☐	A description of any assumptions or corrections, such as tests of normality and adjustment for multiple comparisons
☐	☒	A full description of the statistical parameters including central tendency (e.g. means) or other basic estimates (e.g. regression coefficient) AND variation (e.g. standard deviation) or associated estimates of uncertainty (e.g. confidence intervals)
☐	☒	For null hypothesis testing, the test statistic (e.g. *F*, *t*, *r*) with confidence intervals, effect sizes, degrees of freedom and *P* value noted<br>*Give P values as exact values whenever suitable.*
☒	☐	For Bayesian analysis, information on the choice of priors and Markov chain Monte Carlo settings
☒	☐	For hierarchical and complex designs, identification of the appropriate level for tests and full reporting of outcomes
☐	☒	Estimates of effect sizes (e.g. Cohen's *d*, Pearson's *r*), indicating how they were calculated

Our web collection on statistics for biologists contains articles on many of the points above.

Software and code

Policy information about availability of computer code

Data collection	Confocal mages were acquired below pixel saturation using Confocal Laser Scanning Microscope Leica TCS SP8 (Leica microsystem) or Nikon Inverted confocal spinning-disk microscope or a Plan-Apochromat 63x or 100×/1.4 oil objective on a Zeiss LSM800 or LSM880 confocal system equipped with an AiryScan module and controlled by the Zen blue software; FACS analysis of Sytox Green uptake was performed using BD FACS Celesta™ Cell Analyzer (BD Bioscience); for FLIM-FRET analysis, samples were excited using a pulsed laser (femtosecond Ti:Sa laser, Chameleon VISION 2 from Coherent, set at 900 nm). Photons were temporally collected using a single-photon sensitive detector (PMA-Hybrid 40; Picoquant) combined with a single photon counting module (TimeHarp 260; Picoquant).
Data analysis	Image J-based Fiji, Fiji co-localization plug-in "Coloc2", "Analyze particles" tool of Fiji, FlowJo 10.8.1 software, GraphPad Prism (GraphPad Software). FLIM data analysis was performed using SymPhoTime 64 (Picoquant).

For manuscripts utilizing custom algorithms or software that are central to the research but not yet described in published literature, software must be made available to editors and reviewers. We strongly encourage code deposition in a community repository (e.g. GitHub). See the Nature Portfolio guidelines for submitting code & software for further information.

Data

Policy information about availability of data

All manuscripts must include a data availability statement. This statement should provide the following information, where applicable:

- Accession codes, unique identifiers, or web links for publicly available datasets
- A description of any restrictions on data availability
- For clinical datasets or third party data, please ensure that the statement adheres to our policy

All data is available in the main text, extended data figures or the supplementary materials.

Human research participants

Policy information about studies involving human research participants and Sex and Gender in Research.

Reporting on sex and gender	No human research participants involved
Population characteristics	No human research participants involved
Recruitment	No human research participants involved
Ethics oversight	No human research participants involved

Note that full information on the approval of the study protocol must also be provided in the manuscript.

Field-specific reporting

Please select the one below that is the best fit for your research. If you are not sure, read the appropriate sections before making your selection.

☒ Life sciences ☐ Behavioural & social sciences ☐ Ecological, evolutionary & environmental sciences

For a reference copy of the document with all sections, see nature.com/documents/nr-reporting-summary-flat.pdf

Life sciences study design

All studies must disclose on these points even when the disclosure is negative.

Sample size	For LPS-induced mortality, the sample size was calculated according to the section "Appendix A: Sample size determination" of "Guideline for the care and use of mammals in neuroscience and beharioral research 2003" (PMID: 20669478). In Arfrp1-floxed mice, mortality after LPS injection was estimated at 20%; the expected mortality in myeloid-specific Arfrp1 knockout mice was to be around 70-80%. Based on this, the calculated sample size in each group is 16-18 mice to detect the differences between groups with a statistical power statistical power of 90% and a significance level of 5%. For the rest, no calculation of sample size was performed. A minimum of 3 biologically replicates were measured and analyzed. All the experiments were repeated for at least 3 times with similar results.
Data exclusions	No data exclusions was performed.
Replication	All the experiments have been repeated for at least 3 times. All the attempts of replication were successful.
Randomization	For in vitro experiment, cells from the same pool were randomly split into separate wells and subjected to indicated treatments. For in vivo experiments, mice with the same gender were randomly grouped for treatments. Each group contained similar number of males and females to avoid gender-based effects. The results from both males and females in the same group were pooled and analyzed.
Blinding	No subjective decision making is required for the experiments shown. Investigators were not blinded. There was no need for blinding.

Reporting for specific materials, systems and methods

We require information from authors about some types of materials, experimental systems and methods used in many studies. Here, indicate whether each material, system or method listed is relevant to your study. If you are not sure if a list item applies to your research, read the appropriate section before selecting a response.

Materials & experimental systems

n/a	Involved in the study	
☐	☒	Antibodies
☐	☒	Eukaryotic cell lines
☒	☐	Palaeontology and archaeology
☐	☒	Animals and other organisms
☒	☐	Clinical data
☒	☐	Dual use research of concern

Methods

n/a	Involved in the study	
☒	☐	ChIP-seq
☐	☒	Flow cytometry
☒	☐	MRI-based neuroimaging

Antibodies

Antibodies used

Antibodies used in this study for immunofluorescence analysis are: goat anti-human IL-1β antibody (AF-201-NA, R&D Systems; 1/1000); mouse anti-human Caspase1 antibody (06-503, Merck Millipore; 1/1000); rabbit anti-mouse IL-1β antibody (5129-100, BioVision; 1/1000); mouse anti-mouse Caspase1 p20 (AG-20B-0042-C100, AdipoGen; 1/1000); rabbit anti-ASC antibody (sc-22514, Santa Cruz Biotechnology; 1/1000), mouse anti-NLRP3 antibody (G-20B-0014-C100, AdipoGen; 1/3000); rabbit anti-GAPDH (G9545, Sigma-Aldrich; 1/5000); mouse anti-Tubulin (T9026, Sigma-Aldrich; 1/5000); rabbit anti-ARFRP1 (PA5-50606, ThermoFisher Scientific; 1/2000) and mouse anti-Flag antibody (F1804, Sigma-Aldrich; 1/5000); rabbit anti-OSBP (11096-1-AP, Proteintech; 1/2000); mouse anti-PI4KII alpha (sc-390026, Santa Cruz Biotechnology; 1/1000); rabbit anti-PI4KIIβ (A17719, ABclonal; 1/1000) and mouse anti-PI4KIIIβ (611816, BD Bioscience; 1/1000). Rabbit polyclonal antibodies against VAPA (; 1/6000) and VAPB (1/4000) were generated as described in Venditti et al, 2019 (PMID: 30659099). HRP-conjugated rabbit anti-goat IgG (31402, ThermoFisher Scientific, 1/10000), HRP-conjugated goat anti-rabbit IgG (111-035-144, Jackson ImmunoResearch, 1/10000); goat anti-mouse IgG (31430, ThermoFisher Scientific, 1/10000).
Antibodies used in this study for immunoblotting analysis are: Rabbit anti-TGN46 (13573-1-AP, ProteinTech, 1/300); Sheep anti-human TGN46 (AHP500G, Bio-rad, 1/50); Sheep anti-TGN38 (AHP499G, Bio-rad, 1/50); Rabbit anti-GCC2 (HPA035849, Sigma-Aldrich, 1/200); Rabbit anti-GCC1 (HPA021323, Sigma-Aldrich, 1/100); Mouse anti-human p230 (611280, BD Bioscience, 1/100); Mouse anti-Golgin97 (A-21270, ThermoFisher Scientific, 1/100); Mouse anti-EEA1 (610456, BD Bioscience, 1/100); Rabbit anti-EEA1 (3288S, Cell Signaling Technology, 1/100); Mouse anti-PI4P (Z-P004, Echelon Biosciences, 1/100); Rabbit anti-Golgin97 (Home-made, PMID30659099, 1/100); Alexa Fluor488 goat anti-mouse IgG (A11029, ThermoFisher Scientific, 1/1000); Alexa Fluor594 goat anti-mouse IgG (A11005, ThermoFisher Scientific, 1/1000); Alexa Fluor488 goat anti-mouse IgM (A21042, ThermoFisher Scientific, 1/1000); Alexa Fluor594 goat anti-mouse IgM (A11029, ThermoFisher Scientific, 1/1000); Alexa Fluor488 goat anti-rabbit IgG (A11034, ThermoFisher Scientific, 1/1000); Alexa Fluor594 goat anti-rabbit IgG (A11037, ThermoFisher Scientific, 1/1000); Alexa Fluor647 goat anti-rabbit IgG (A21246, ThermoFisher Scientific, 1/1000) and Alexa Fluor488 donkey anti-sheep IgG (A11015, ThermoFisher Scientific, 1/1000).

Validation

Goat anti-human IL-1β antibody (AF-201-NA, R&D Systems); mouse anti-human Caspase-1 antibody (06-503, Merck Millipore); rabbit anti-mouse IL-1β antibody (5129-100, BioVision); mouse anti-mouse Caspase-1 p20 (AG-20B-0042-C100, AdipoGen); rabbit anti-ASC antibody (sc-22514, Santa Cruz Biotechnology), mouse anti-NLRP3 antibody (G-20B-0014-C100, AdipoGen) were widely used in inflammasome-related studies, including our previous study (PMID: 28716882); rabbit anti-VAPA (home-made); rabbit anti-VAPB (home-made); rabbit anti-OSBP (11096-1-AP, Proteintech); mouse anti-PI4KII alpha(sc-390026, Santa Cruz Biotechnology), rabbit anti-PI4KII beta (A17719, ABclonal) and mouse anti-PI4KIIIβ antibody (611816, BD Bioscience) were validated in this study in Fig. 3c, 3d, Extended Data Fig. 3b, 3e; rabbit anti-ARFRP1 (PA5-50606, ThermoFisher Scientific); rabbit anti-GCC2 antibody (HPA035849, Sigma-Aldrich); rabbit anti-GCC1 antibody (HPA021323, Sigma-Aldrich); mouse anti-human p230 (611280, BD Bioscience) and mouse anti-Golgin97 (A-21270, ThermoFisher Scientific) were validated in previous study (PMID: 31575603); mouse anti-EEA1 (610456, BD Bioscience), rabbit anti-EEA1 (3288S, Cell Signaling Technology), sheep anti-human TGN46 (AHP500GT, Bio-rad) and rabbit anti-Golgin97 were validated by previous studies (including PMID: 30659099); mouse anti-PI4P (Z-P004, Echelon Biosciences) was validated by previous studies (including PMID: 30659099 and PMID: 19508231).

Eukaryotic cell lines

Policy information about cell lines and Sex and Gender in Research

Cell line source(s)

THP-1 cells was from ATCC; HEK293t cells (ATCC, CRL-3216) was from DKFZ Heidelberg; Immortalized bone marrow-derived macrophages (iBMDMs) cell line was obtained from Dr. Eicke Latz (University of Bonn, Germany). mApple-tagged RAB5 stably-expressing HeLa cell line was provided by Dr. Anne Spang (BIOZENTRUM, University of Basel, Switzerland). HeLa WT and HeLa VAP dKO cell lines10 were a generous gift from Dr. Pietro De Camilli (Yale University School of Medicine, New Haven, CT).

Authentication

THP-1 cells and HEK293t cells have been authenticated using Short Tandem Repeat (STR) performed by LGC Standards, UK. HeLa cells and iBMDMs were not authenticated.

Mycoplasma contamination

All the cells used in this study were tested Mycoplasma-negative.

Commonly misidentified lines
(See ICLAC register)

No misidentified cell lines were used in this study.

Animals and other research organisms

Policy information about studies involving animals; ARRIVE guidelines recommended for reporting animal research, and Sex and Gender in Research

Laboratory animals	Mice were housed under specific pathogen-free conditions with controlled temperature (19-23°C) and humidity (40-60%) on a 12-h light/dark cycle with unrestricted access to water and standard laboratory chow. Arfrp1-floxed and myeloid-specific Arfrp1 knockout mice on C57BL/6J background at age 6-8 weeks-old were used.
Wild animals	No wild animals were used in this study.
Reporting on sex	Both males and females were used in this study.
Field-collected samples	No field-collected samples were used in this study.
Ethics oversight	Maintenance and animal experimentation were in accordance with the local ethical committee (Com'Eth) in compliance with the European legislation on care and use of laboratory animals (La cellule AFiS (Animaux utilisés à des Fins Scientifiques): APAFIS#30865-2021040115389039 v3)

Note that full information on the approval of the study protocol must also be provided in the manuscript.

Flow Cytometry

Plots

Confirm that:

☒ The axis labels state the marker and fluorochrome used (e.g. CD4-FITC).

☒ The axis scales are clearly visible. Include numbers along axes only for bottom left plot of group (a 'group' is an analysis of identical markers).

☒ All plots are contour plots with outliers or pseudocolor plots.

☒ A numerical value for number of cells or percentage (with statistics) is provided.

Methodology

Sample preparation	After treatment, cells were detached with 5 mM EDTA and incubated with SYTOX™ Green (S7020, ThermoFisher Scientific) at 1/8000 dilution.
Instrument	BD FACS Celesta™ Cell Analyzer (BD Bioscience)
Software	FlowJo 10.8.1 Software
Cell population abundance	80~90% of VAP dKO, OSBP KO, ARFRP1 or SYS1 KO THP-1 cells became SYTOX Green-positive when treated with 1 µg/ml LPS or Pam3CSK4; while WT cells showed less than 10% of SYTOX Green-positive cells.
Gating strategy	Forward and side scatter and SYTOX Green signal-based gating

☒ Tick this box to confirm that a figure exemplifying the gating strategy is provided in the Supplementary Information.

March 2021
