# Supplementary material for: Distinct changes in endosomal composition promote NLRP3 inflammasome activation
Source: Nat Immunol. 2022 Nov 28;24(1):30–41. doi: 10.1038/s41590-022-01355-3 (PMC9810532; doi:10.1038/s41590-022-01355-3)
Supplement: Source Data Fig. 3 — Unprocessed western blots. [file 41590_2022_1355_MOESM8_ESM.pdf]

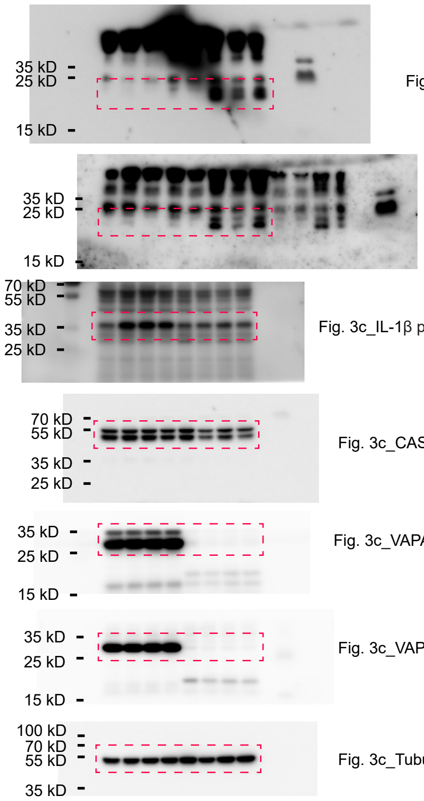


Fig. 3c_IL-1β p17 (Sup)

Fig. 3c_CASP1 p20 (Sup)

Fig. 3c_IL-1β p31 (Lys)

Fig. 3c_CASP1 p45 (Lys)

Fig. 3c_VAPA (Lys)

Fig. 3c_VAPB (Lys)

Fig. 3c_Tubulin (Lys)

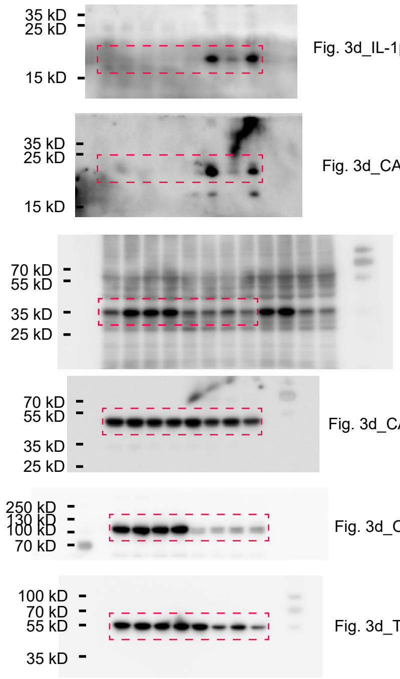


Fig. 3d_IL-1β p17 (Sup)

Fig. 3d_CASP1 p20 (Sup)

Fig. 3d_IL-1β p31 (Lys)

Fig. 3d_CASP1 p45 (Lys)

Fig. 3d_OSBP (Lys)

Fig. 3d_Tubulin (Lys)

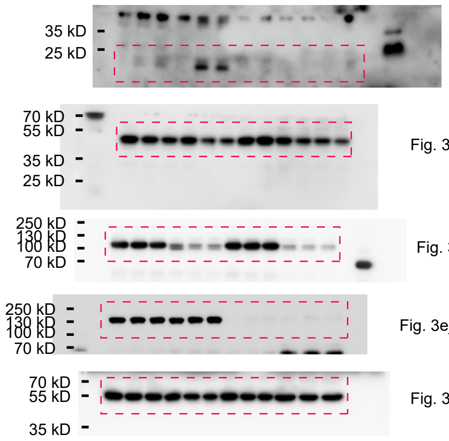


Fig. 3e_CASP1 p20 (Sup)

Fig. 3e_CASP1 p45 (Lys)

Fig. 3e_OSBP (Lys)

Fig. 3e_NLRP3 (Lys)

Fig. 3e_Tubulin (Lys)

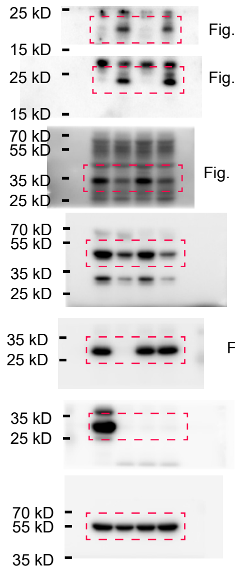


Fig. 3f_IL-1β p17 (Sup)

Fig. 3f_CASP1 p20 (Sup)

Fig. 3f_IL-1β p31 (Lys)

Fig. 3f_CASP1 p45 (Lys)

Fig. 3f_VAPA (Lys)

Fig. 3f_VAPB (Lys)

Fig. 3f_Tubulin (Lys)
