# Supplementary material for: Distinct changes in endosomal composition promote NLRP3 inflammasome activation
Source: Nat Immunol. 2022 Nov 28;24(1):30–41. doi: 10.1038/s41590-022-01355-3 (PMC9810532; doi:10.1038/s41590-022-01355-3)
Supplement: Source Data Fig. 7 — Unprocessed western blots. [file 41590_2022_1355_MOESM10_ESM.pdf]

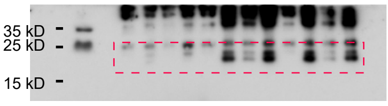

Fig. 7b_IL-1β p17 (Sup)

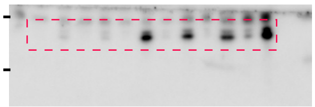

Fig. 7b_CASP1 p20 (Sup)

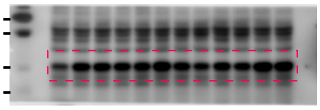

Fig. 7b_IL-1β p31 (Lys)

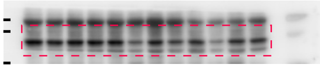

Fig. 7b_CASP1 p45 (Lys)

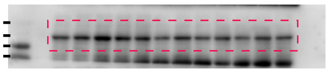

Fig. 7b_NLRP3 (Lys)

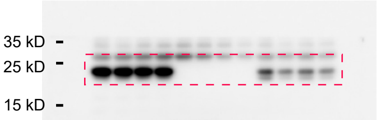

Fig. 7b_ARFRP1 (Lys)

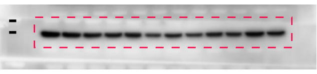

Fig. 7b_Tubulin (Lys)

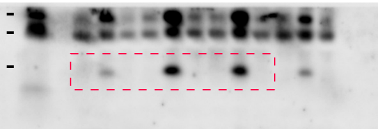

Fig. 7d_IL-1β p17 (Sup)

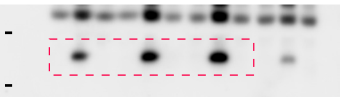

Fig. 7d_CASP1 p20 (Sup)

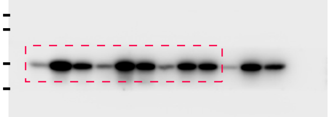

Fig. 7d_IL-1β p31 (Lys)

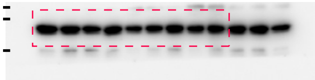

Fig. 7d_CASP1 p45 (Lys)

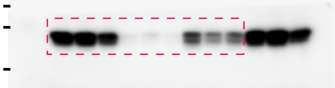

Fig. 7d_ARFRP1 (Lys)

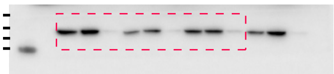

Fig. 7d_NLRP3 (Lys)

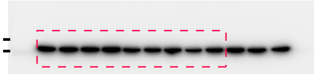

Fig. 7d_Tubulin (Lys)

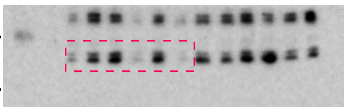

Fig. 7g(left)_CASP1 p20 (Sup)

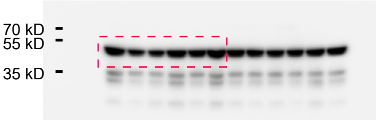

Fig. 7g(left)_CASP1 p45 (Lys)

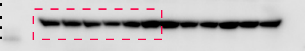

Fig. 7g(left)_NLRP3 (Lys)

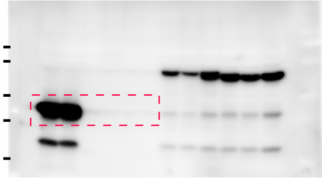

Fig. 7g(left)_GFP (Lys)

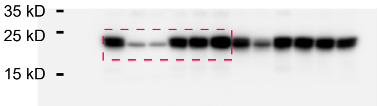

Fig. 7g(left)_ARFRP1 (Lys)

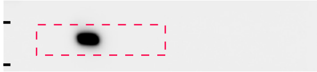

Fig. 7g(left)_Flag (Lys)

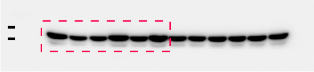

Fig. 7g(left)_Tubulin (Lys)

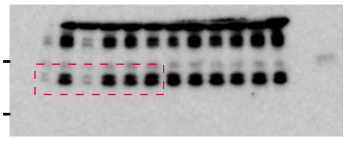

Fig. 7g(right)_CASP1 p20 (Sup)

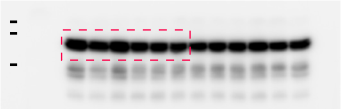

Fig. 7g(right)_CASP1 p45 (Lys)

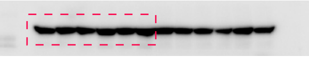

Fig. 7g(right)_NLRP3 (Lys)

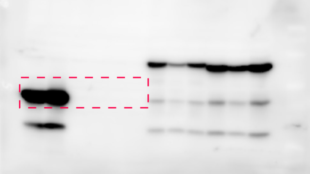

Fig. 7g(right)_GFP (Lys)

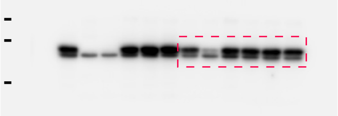

Fig. 7g(right)_ARFRP1 (Lys)

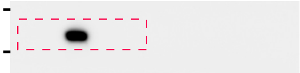

Fig. 7g(right)_Flag (Lys)

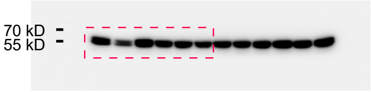

Fig. 7g(right)_Tubulin (Lys)

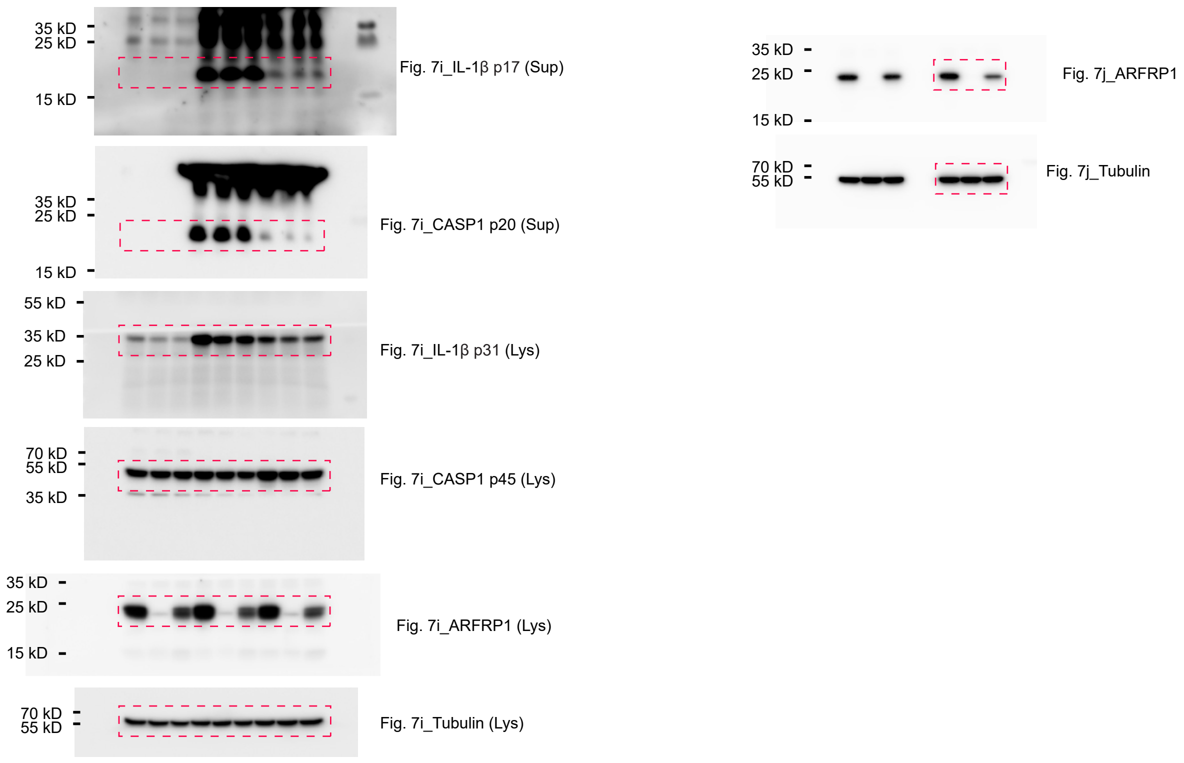
35 kD
25 kD
15 kD
Fig. 7i_IL-1β p17 (Sup)
35 kD
25 kD
15 kD
Fig. 7i_CASP1 p20 (Sup)
55 kD
35 kD
25 kD
Fig. 7i_IL-1β p31 (Lys)
70 kD
55 kD
35 kD
Fig. 7i_CASP1 p45 (Lys)
35 kD
25 kD
15 kD
Fig. 7i_ARFRP1 (Lys)
70 kD
55 kD
Fig. 7i_Tubulin (Lys)
35 kD
25 kD
15 kD
Fig. 7j_ARFRP1
70 kD
55 kD
Fig. 7j_Tubulin
