# Supplementary material for: Distinct changes in endosomal composition promote NLRP3 inflammasome activation
Source: Nat Immunol. 2022 Nov 28;24(1):30–41. doi: 10.1038/s41590-022-01355-3 (PMC9810532; doi:10.1038/s41590-022-01355-3)
Supplement: Source Data Fig. 8 — Unprocessed western blots. [file 41590_2022_1355_MOESM12_ESM.pdf]

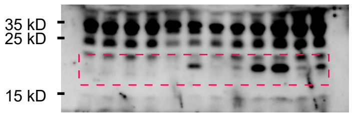

Fig. 8a_IL-1β p17 l.e. (Sup)

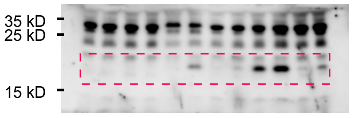

Fig. 8a_IL-1β p17 s.e. (Sup)

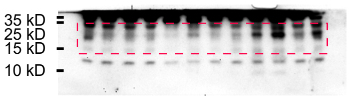

Fig. 8a_CASP1 p20 l.e. (Sup)

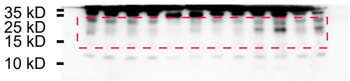

Fig. 8a_CASP1 p20 s.e. (Sup)

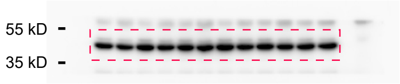

Fig. 8a_CASP1 p45 (Lys)

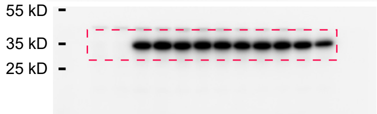

Fig. 8a_IL-1β p31 (Lys)

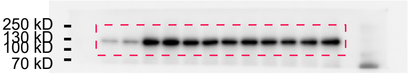

Fig. 8a_NLRP3 (Lys)

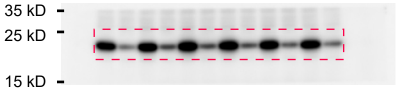

Fig. 8a_ARFRP1 (Lys)

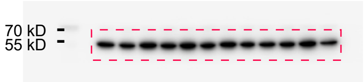

Fig. 8a_Tubulin (Lys)
