# Supplementary material for: Distinct changes in endosomal composition promote NLRP3 inflammasome activation
Source: Nat Immunol. 2022 Nov 28;24(1):30–41. doi: 10.1038/s41590-022-01355-3 (PMC9810532; doi:10.1038/s41590-022-01355-3)
Supplement: Source Data Extended Data Fig. 3 — Unprocessed western blots. [file 41590_2022_1355_MOESM13_ESM.pdf]

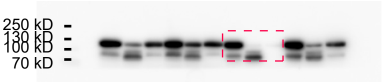

Extended Data Fig. 3b_PI4KIIIβ

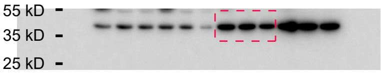

Extended Data Fig. 3b_GAPDH

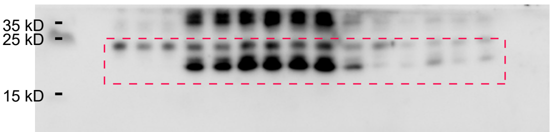

Extended Data Fig. 3e_IL-1β p17 (Sup)

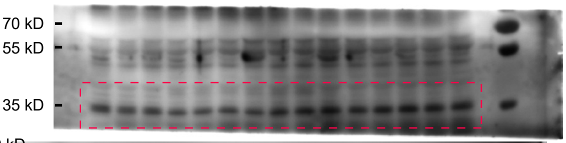

Extended Data Fig. 3e_IL-1β p31 (Lys)

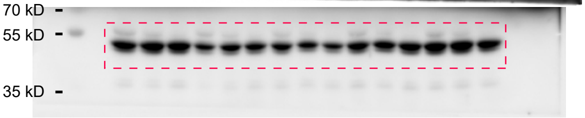

Extended Data Fig. 3e_CASP1 p45 (Lys)

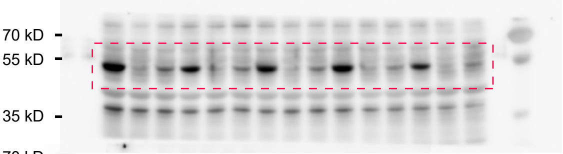

Extended Data Fig. 3e_PI4KIIα (Lys)

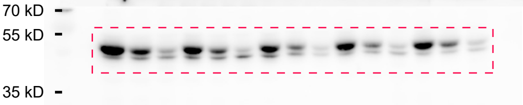

Extended Data Fig. 3e_PI4KIIβ (Lys)

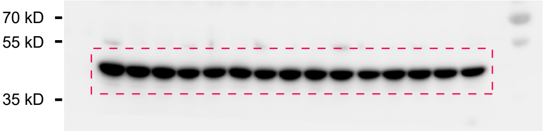

Extended Data Fig. 3e_Actin (Lys)
