# Supplementary figures and images for: Distinct changes in endosomal composition promote NLRP3 inflammasome activation
Source: Nat Immunol. 2022 Nov 28;24(1):30–41. doi: 10.1038/s41590-022-01355-3 (PMC9810532; doi:10.1038/s41590-022-01355-3)

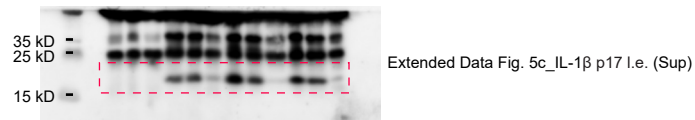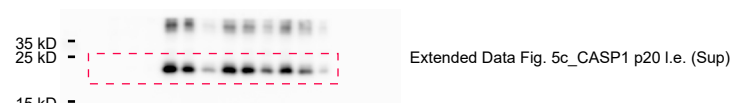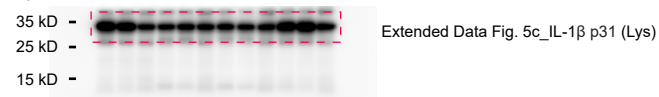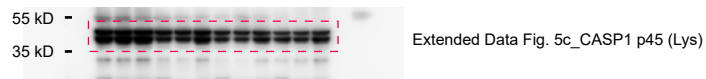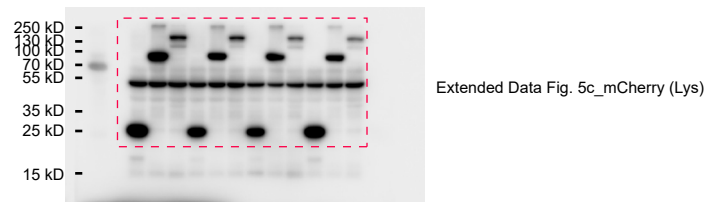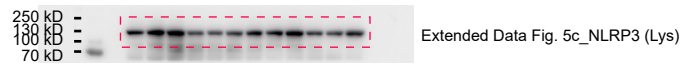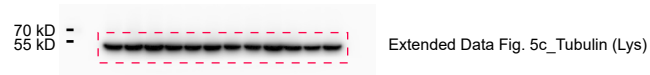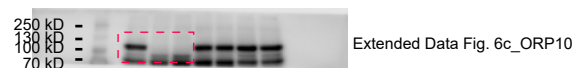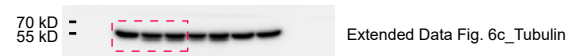

Supplement: Source Data Extended Data Fig. 5 — Unprocessed western blots. [file 41590_2022_1355_MOESM14_ESM.pdf]
